# Combined inhibition of Notch and FLT3 produces synergistic cytotoxic effects in FLT3/ITD^+^ acute myeloid leukemia

**DOI:** 10.1038/s41392-020-0108-z

**Published:** 2020-03-13

**Authors:** Dan Li, Tongjuan Li, Zhen Shang, Lei Zhao, Qian Xu, Jiaqi Tan, Yun Qin, Yuanyuan Zhang, Yang Cao, Na Wang, Liang Huang, Xiaojian Zhu, Kuangguo Zhou, Liting Chen, Chunrui Li, Ting Xie, Yi Yang, Jue Wang, Jianfeng Zhou

**Affiliations:** 1 Department of Hematology, Tongji Hospital, Tongji Medical College, Huazhong University of Science and Technology, Wuhan, Hubei 430030 China; 2 Division of Pathology and Experimental Hematology and Cancer Biology, Cincinnati Children’s Hospital Medical Center, Cincinnati, OH 45248 USA; 3 Immunotherapy Research Center for Hematologic Diseases of Hubei Province, Wuhan, Hubei 430030 China; 4 Department of geriatrics, Tongji Hospital, Tongji Medical College, Huazhong University of Science and Technology, Wuhan, Hubei 430030 China; 5 Medical Research Institute, Wuhan University, Wuhan, 430071 China

**Keywords:** Haematological cancer, Molecular medicine

## Abstract

Internal tandem duplication (ITD) mutations of FMS-like tyrosine kinase-3 (FLT3) are the most frequent genetic alterations in acute myeloid leukemia (AML) and predict a poor prognosis. FLT3 tyrosine kinase inhibitors (TKIs) provide short-term clinical responses, but the long-term prognosis of FLT3/ITD^+^ AML patients remains poor. Notch signaling is important in numerous types of tumors. However, the role of Notch signaling in FLT3/ITD^+^ AML remains to be elucidated. In the current study, we found that Notch signaling was activated upon FLT3-TKI treatment in FLT3/ITD^+^ cell lines and primary cells. As Notch signaling can be blocked by γ-secretase inhibitors (GSIs), we examined the combinatorial antitumor efficacy of FLT3-TKIs and GSIs against FLT3/ITD^+^ AML and explored the underlying molecular mechanisms. As a result, we observed synergistic cytotoxic effects, and the treatment preferentially reduced cell proliferation and induced apoptosis in FLT3/ITD^+^ AML cell lines and in primary AML cells. Furthermore, the combination of FLT3-TKI and GSI eradicated leukemic cells and prolonged survival in an FLT3/ITD^+^ patient-derived xenograft AML model. Mechanistically, differential expression analysis suggested that CXCR3 may be partially responsible for the observed synergy, possibly through ERK signaling. Our findings suggest that combined therapies of FLT3-TKIs with GSI may be exploited as a potential therapeutic strategy to treat FLT3/ITD^+^ AML.

## Introduction

Internal tandem duplication (ITD) in the juxtamembrane portion of FMS-like tyrosine kinase-3 (FLT3) is one of the most prevalent molecular alterations in acute myeloid leukemia (AML).^1^ FLT3-ITD mutations cause constitutive activation of FLT3 signaling and its downstream signaling pathways, including MAPK/ERK, JAK/STAT5, and PI3K/AKT, resulting in uncontrolled proliferation, inhibition of differentiation, and reduction of apoptosis in AML cells.^2^ AML patients with FLT3-ITD mutations present with high relapse rates and poor overall survival.^3,4^

Although continued efforts are being made to develop potent FLT3 tyrosine kinase inhibitors (TKIs), FLT3-ITD mutations are still associated with a dismal prognosis in AML.^5^ Thus, a number of combination therapies are being explored. TKIs in combination with inhibitors targeting glutaminolysis,^6^ PI3Kd,^7^ ERK signaling^8^, or Wnt/β-catenin^9^ have shown synergistic lethal effects with the potential to overcome drug resistance. Signal transduction pathways, such as WNT/β-catenin, retinoic acid, Notch, and Hedgehog, have been identified as important for maintaining leukemia stem/progenitor cell (LSC) self-renewal in AML^10^ and are therefore considered potential therapeutic targets. The WNT/β-catenin inhibitor BC2059 demonstrates significant killing efficiency against the CD34^+^CD38^−^Lin^−^ stem cell/progenitor cell population in FLT3/ITD^+^ AML.^11^ Retinoic acid combined with FLT3-TKIs can effectively remove FLT3/ITD^+^ LSCs and prolong survival in FLT3/ITD^+^ AML xenograft models.^12^ However, the effects of Notch signaling in FLT3/ITD^+^ AML remain to be elucidated.

Notch signaling is an evolutionarily conserved pathway that plays an important role in a wide variety of cellular processes, including differentiation, proliferation, apoptosis, and stem cell maintenance.^13^ Notch signaling is initiated by the interaction of Notch ligands and receptors on adjacent cells, which further triggers two proteolytic cleavage events. The first cleavage releases a functional extracellular domain (NECD); the second cleavage, mediated by γ-secretase, releases the intracellular domain (NICD) into the cytoplasm.^14^ The NICD then translocates to the nucleus, binds to the transcription factor CBF/Su (H)/LAG-2 (CSL), and recruits Mastermind-like protein 1 and p300/CBP to induce transcription of Notch target genes, including *Hes1, p21, Akt, cyclin D1*, and *mTOR*.^15^ Gamma secretase inhibitors (GSIs), a class of small molecules that suppress the cleavage of γ‐secretase substrates, can effectively block Notch signaling and exhibit potent antitumor activities in several cancers.^16^ Recent studies have shown that the combination of GSIs with traditional chemicals has synergistic effects and may overcome drug resistance.^17,18^

In view of the potential of Notch inhibition in various tumors, we focused on its unique role in AML. In this context, we further explored the rationale of simultaneously targeting the FLT3 and Notch pathways with a TKI and a GSI in FLT3/ITD^+^ AML.

## Results

### Modulation of Notch signaling by FLT3-TKIs

**Fig. 1:**
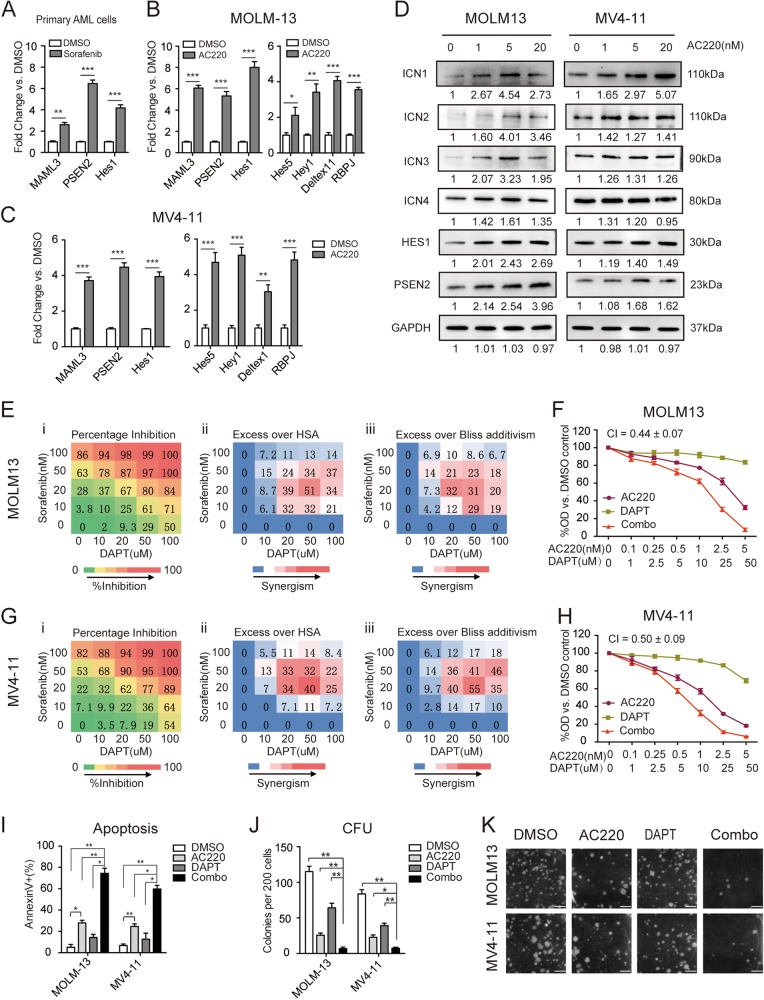
FLT3-TKIs mediate the upregulation of Notch signaling, and combined treatment of FLT3-TKIs with DAPT triggers synergistic cytotoxic effects on FLT3/ITD^+^ cells. **a** The expression levels of *MAML3*, *PSEN2*, and *Hes1* were measured in triplicate by qPCR relative to *GAPDH* in samples obtained from FLT3/ITD^+^ patients (*n* = 3) treated with DMSO or sorafenib. **b** MOLM13 cells and **c** MV4-11 cells were treated with AC220 (2.5 nM) for 12 h, and the mRNA expression levels of *MAML3*, *PSEN2*, *Hes1*, *Hes5*, *Hey1, Deltex1, and RBPJ* were measured in triplicate by qPCR relative to *GAPDH* levels. **d** The expression of ICN1, ICN2, ICN3, ICN4, PSEN2, and HES1 was determined by immunoblotting after treatment with AC220 at the indicated concentrations for 12 h in both MOLM13 and MV4-11 cells. GAPDH was used as a loading control. **e** (i) The percentage inhibition of MOLM13 cell proliferation relative to that of untreated cells is shown. Cells were treated at the indicated concentrations of sorafenib (0–100 nM) and DAPT (0–100 µM), and cell viability was determined by the CCK-8 assay following 72 h of treatment. The average of three measurements is shown. The color of the squares also indicates the level of growth inhibition. (ii) Differences in the percentage growth inhibition between the combination treatment and either sorafenib or DAPT treatment alone, whichever had a stronger effect. HSA highest single agent. (iii) Excess over Bliss additivism was determined by the difference between the observed and the predicted percentage inhibition of the combined treatment. The Bliss additivism model predicting the combined response *C* for two single compounds with effects *A* and *B* is *C* = *A* + *B* − *A* × *B*, where *A* and *B* are the percentage inhibition of single agents A and B. The difference reflects the magnitude of synergism, as shown by the scale bar. **f** MOLM13 cells were treated with AC220 (0–5 nM) for 72 h, either alone or in combination with DAPT (0–50 µM), and cell proliferation was measured in triplicate by the CCK-8 assay. Data represent the average ±SD. **g** (i–iii) and **h** Results obtained from MV4-11 cells. **i** MOLM13 and MV4-11 cells were treated with AC220 (2.5 nM) and/or DAPT (25 µM). Apoptosis was measured by Annexin V staining at 48 h. **j** CFU numbers 14 days after plating of 2 × 10^2^ MOLM13 or MV4-11 cells in culture medium containing AC220 (1 nM) and/or DAPT (10 µM). **k** Representative images of the colony-forming assay are shown. Images were obtained using the white mode of ChemiDoc XRS + Imaging System (Bio-Rad). Scale bar, 20 mm. Data represent the average of three independent experiments ±SD. (*P < 0.05; **P < 0.01, ***P < 0.001).

We first investigated the effects of TKI treatment on the Notch signaling response in FLT3/ITD^+^ AML. The expression of key factors in Notch signaling, including *MAML3*, *PSEN2*, and the downstream target gene *Hes1*, was significantly increased after sorafenib treatment in three AML patients carrying the FLT3-ITD mutation (Fig. 1a). The same increase in the RNA expression of *MAML3*, *PSEN2*, and *Hes1*, as well as the Notch downstream target genes *Hes5, Hey1, Deltex1, and RBPJ*, was observed upon treatment with AC220 of two human FLT3/ITD^+^ AML cell lines (MOLM13 and MV4-11) (Fig. 1b, c). The activation of Notch signaling was validated by western blotting of ICN1, ICN2, ICN3, ICN4, PSEN2, and HES1 (Fig. 1d). As Notch signaling is generally silenced in AML at baseline,^19^ we hypothesized that Notch activation after FLT3-TKI treatment may be a cellular feedback mechanism to compensate for drug-induced cytotoxicity, and Notch inhibition may enhance the effects of FLT3-TKIs.

### Synergistic effects of FLT3-TKIs and GSIs in FLT3/ITD^+^ cell lines

To assess the effects of combination therapy, MOLM13 and MV4-11 cells were treated with increasing concentrations of TKI-sorafenib alone, the γ-secretase inhibitor (GSI) DAPT {N-[N-(3,5-difluorophenacetyl)-l-alanyl]-S-phenylglycine t-butyl ester alone, or sorafenib plus DAPT. DAPT treatment resulted in decreased cleaved intracellular Notch receptors, as expected (Supplemental Fig. S1). Proliferation of the FLT3/ITD^+^ cell lines was sensitive to sorafenib, while DAPT alone resulted in a slight change in the rate of proliferation. Synergism between sorafenib and DAPT could be demonstrated using the HSA model and Bliss model^20,21^ in both MOLM13 and MV4-11 cells (Fig. 1e, g). Similar combinatorial effects were observed when sorafenib was replaced with AC220. As shown in Fig. 1f, h, the combination index (CI) value was <1, indicating a synergistic antiproliferative effect when FLT3/ITD^+^ cells were treated with both GSI and TKI. In contrast, when FLT3/WT cell lines (THP-1 and OCI-AML3) were treated with higher concentrations of AC220 alone or in combination with DAPT, no synergistic effects were observed (Supplemental Fig. S2a, b).

To further explore the synergistic effects of combined FLT3 TKIs and DAPT treatment in FLT3/ITD^+^ leukemia cells, apoptosis assays were performed using MOLM13 and MV4-11 cells. A fixed concentration of each drug (2.5 nM AC220 and 25 µM DAPT) was chosen to assess the combinatorial effects on apoptosis. After 48 h of treatment with AC220 and/or DAPT, the combinatorial regimen was confirmed to exert synergistic proapoptotic effects compared with either agent alone (Fig. 1i). To confirm the synergistic effects of combining FLT3 TKIs and GSIs in FLT3/ITD^+^ cells, an alternative TKI (sorafenib) and GSI (RO4929097) were tested. Similar synergistic effects were recorded for sorafenib plus DAPT and for AC220 plus RO4929097 in FLT3/ITD^+^ cells (Supplemental Fig. S2c, d), whereas no combinatorial proapoptotic effects were observed in FLT3/WT cell lines (THP-1 and OCI-AML3) when treated with even higher concentrations of AC220 (250 nM) alone or in combination with DAPT (25 µM) (Supplemental Fig. S2e).

As Notch signaling plays an important role in stem cell maintenance, the long-term effects of Notch inhibitors on leukemia cells were evaluated by colony-forming assays (CFU). Leukemia cells were treated with AC220 (1 nM for FLT3/ITD^+^ cells and 100 nM for FLT3/WT cells) and/or DAPT (10 µM) for 14 days, which were lower concentrations than those in the apoptosis assays. Although treatment with AC220 or DAPT alone led to a moderate reduction, clonogenicity was strikingly decreased in response to the combinatorial regimen in the FLT3/ITD^+^ cell lines MOLM13 and MV4-11 (Fig. 1j, k, Supplemental Fig. S9). In contrast, only DAPT decreased the clonogenicity of the FLT3/WT cell lines THP-1 and OCI-AML3, with no further reduction observed in response to combination treatment (Supplemental Fig. S2f).

### Combinatorial effect of AC220 with Notch inhibition in FLT3/ITD cells and FLT3/ITD^+^ patient blast samples

To better address the specificity of the synergy between TKIs and GSIs, we generated isogenic clones carrying the FLT3/ITD mutation in the SKM-1 leukemia cell line, with a 21-bp ITD fragment knocked in using the CRISPR/Cas9 system (Supplemental Fig. S3a). qPCR and western blotting demonstrated a significant increase in FLT3 expression and phosphorylation of classical FLT3 downstream signaling, as evidence of successful knock-in (Supplemental Fig. S3b, c). Among three FLT3/ITD knock-in clones, clone 02, also named SKM-1-1D5, was chosen for subsequent experiments. In SKM-1-1D5 cells, aberrant activation of FLT3/ITD-associated pathways was decreased upon AC220 treatment, further confirming that the ITD mutation was successfully knocked in and functional at the posttranscriptional level (Supplemental Fig. S3d). As one of the most prominent characteristics of the FLT3-ITD mutation is the promotion of cell proliferation and resistance to apoptosis, the proliferation and apoptosis of SKM-1-1D5 cells were tested. As expected, SKM-1-1D5 cells displayed a stronger proliferative capacity, less apoptosis under starvation conditions, and greater colony-forming ability in comparison to the isogenic counterpart SKM-1-WT cells (Supplemental Fig. S3e-g). Overall, the SKM-1-1D5 clone could be an appropriate model for research on FLT3/ITD^+^ AML.

**Fig. 2:**
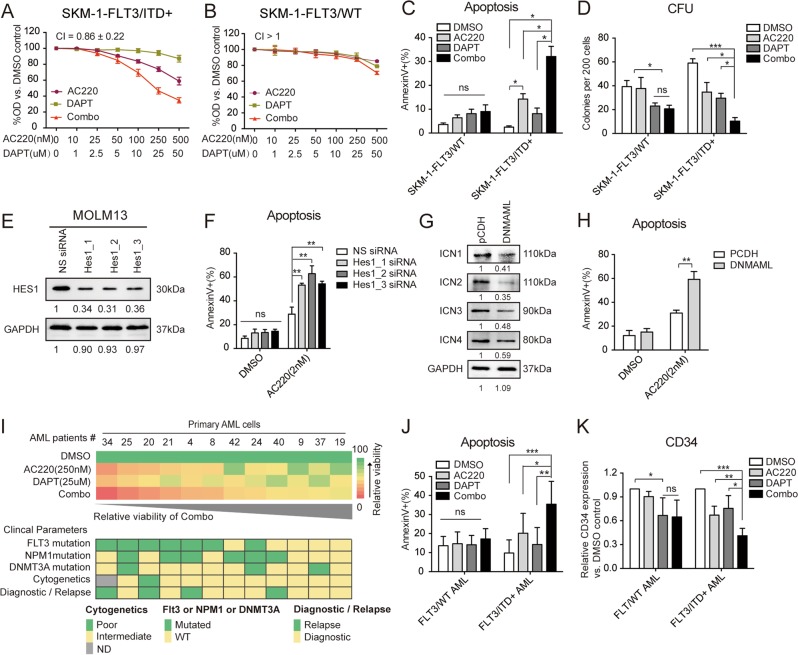
Combinatorial effect of AC220 with Notch inhibition in FLT3/ITD cells and FLT3/ITD^+^ patient blast samples. **a** FLT3/ITD^+^ (SKM-1-1D5) and **b** FLT3/WT (SKM-1-WT) SKM-1 cells were treated in triplicate with AC220 (0-500 nM) for 72 h either alone or in combination with DAPT (0–50 µM), and cell proliferation was measured by the CCK-8 assay. **c** FLT3/ITD^+^ (SKM-1-1D5) and FLT3/WT (SKM-1-WT) SKM-1 cells were treated in triplicate with AC220 (250 nM) and/or DAPT (25 µM) for 48 h, and apoptosis was measured by Annexin V staining. **d** CFU counts at 14 days of FLT3/ITD^+^ (SKM-1-1D5) and FLT3/WT (SKM-1-WT) SKM-1 cells cultured in methylcellulose-based medium containing AC220 (100 nM) and/or DAPT (10 µM). Data represent the average of three independent experiments ±SD. (*P < 0.05; **P < 0.01, ***P < 0.001). **e** Expression of HES1 in MOLM13 cells transfected with non-silencing (NS) siRNA or Hes1-targeting (Hes1_1, Hes1_2, and Hes1_3) siRNAs. **f** Hes1 knockdown and non-silencing control MOLM13 cells were treated with AC220 (2 nM), and apoptosis was measured by Annexin V staining at 48 h. **g** MOLM13 cells infected with lentivirus expressing pCDH or pCDH-DNMAML were selected by puromycin (1 μg/mL) for 1 week. The expression of ICN1, ICN2, ICN3, and ICN4 in purified cells was measured by immunoblotting. GAPDH was used as a loading control. **h** Puromycin-purified MOLM13 cells expressing pCDH or pCDH-DNMAML were subjected to DMSO or AC220 (2 nM) treatment for 48 h. Apoptosis was measured by Annexin V staining. Data represent the average of three independent experiments ± SD. (*P *<* 0.05). **i** Primary AML samples (*n* = 12) were treated with AC220 (250 nM) and/or DAPT (25 μM) for 48 h. Cell viability relative to DMSO control cell viability was measured in triplicate by CCK-8 assay. The corresponding clinical parameters for each AML patient are presented below. Samples were ranked by their sensitivity towards the combined treatment. **j** Apoptosis of treated FLT3/ITD^+^ (*n* = 7) and FLT3/WT (*n* = 5) primary cells was measured in triplicate by Annexin V staining. **k** Surface expression of CD34 in primary blast cells following the indicated treatment, as represented by the relative CD34 proportion vs. the DMSO control. Error bars represent the average ±SD. (*P *<* 0.05; **P *<* 0.01, ***P *<* 0.001).

Therefore, the antiproliferative effects of AC220 (0–250 nM) in combination with or without DAPT (0–25 µM) were tested in SKM-1-WT and SKM-1-1D5 cells. SKM-1-1D5 cells were more sensitive to AC220 treatment, and synergistic effects could only be observed (CI < 1) in SKM-1-1D5 cells when AC220 was combined with DAPT. Both SKM-1-WT and SKM-1-1D5 cells demonstrated little decrease in proliferation in response to DAPT treatment alone (Fig. 2a, b). Combined treatment of AC220 and DAPT also induced increased apoptosis in SKM-1-1D5 cells compared with either drug alone. In contrast, AC220 treatment alone resulted in limited apoptosis in SKM-1-WT cells, while AC220 plus DAPT showed no additive effect (Fig. 2c, Supplemental Fig. S9). Furthermore, the effects of AC220 and/or DAPT on colony formation were assessed. In SKM-1-1D5 cells, AC220 plus DAPT treatment led to a significant reduction in clonogenicity compared with either agent alone. No synergy was observed in SKM-1-WT cells, with only a slight decrease in colony numbers following treatment with DAPT (Fig. 2d). Overall, these results indicated that the synergistic antitumor effects of cotreatment with AC220 and DAPT were restricted to FLT3/ITD-mutated leukemia cells.

To further validate the relevance of Notch signaling inhibition to the observed synergistic effects, small interfering RNA (siRNA) targeting *Hes1* and lentivirus expressing dominant-negative mastermindlike-1 (DNMAML) were used. MOLM13 cells were electroporated with non-silencing (NS) or one of three Hes1-targeting (Hes1_1, Hes1_2, and Hes1_3) siRNAs. Following electroporation, there was a significant decrease in HES1 protein levels compared to the effect of NS siRNA (Fig. 2e). Consequently, upon treatment with AC220, there was an increase in apoptosis of MOLM13 cells transfected with Hes1-targeting siRNAs compared to NS siRNA (Fig. 2f, Supplemental Fig. S9). Similarly, MOLM13 cells were infected with lentivirus expressing vector alone (pCDH) or DNMAML. DNMAML-infected cells exhibited decreased expression of ICN1~4 (Fig. 2g) and significantly increased apoptosis upon AC220 treatment compared to pCDH-infected cells (Fig. 2h, Supplemental Fig. S9). Both experiments showed that Notch inactivation renders FLT3/ITD + MOLM13 cells more sensitive to the FLT3 inhibitor AC220.

We next examined the effects of AC220 combined with DAPT on primary AML cells. Peripheral blood samples from 12 AML patients were collected. Mononuclear cells were isolated and cultured with AC220 (250 nM) and/or DAPT (25 µM) for 48 h. Although these primary cells showed different sensitivities to AC220 and DAPT, stronger inhibition of cell proliferation by the combined treatment was more likely to be observed in specimens from FLT3/ITD^+^ patients (Fig. 2i). Consistent with the cell lines results, a significant increase in Annexin V+ binding was detected following combined treatment with DAPT and AC220 in FLT3/ITD^+^ primary samples compared to either DAPT or AC220 alone (Fig. 2j, Supplemental Fig. S9).

Moreover, we measured the proportion of CD34^+^ cells in primary samples, generally representing leukemia stem cells (LSCs) or normal hematopoietic stem/progenitor cells (HSPCs). Although AC220 or DAPT alone moderately reduced the proportion of CD34^+^ in FLT3/ITD^+^ samples, AC220 plus DAPT could further reduce the CD34^+^ ratio compared with each single agent. In contrast, only DAPT slightly reduced the CD34^+^ proportion of FLT3/WT blasts. Interestingly, the CD34^+^ ratios in healthy donors (*n* = 4) were unaffected by AC220 and/or DAPT at the tested concentrations, indicating that the LSCs were more sensitive to the combined treatment than normal HSPCs (Fig. 2k, Supplemental Fig. S4a). In addition, the apoptosis of peripheral blood mononuclear cells was not affected by DAPT treatment alone at the tested concentrations (Supplemental Fig. S4b).

### The combination of sorafenib and DAPT exhibits therapeutic efficacy in a patient-derived xenograft model of FLT3/ITD^+^ AML

**Fig. 3:**
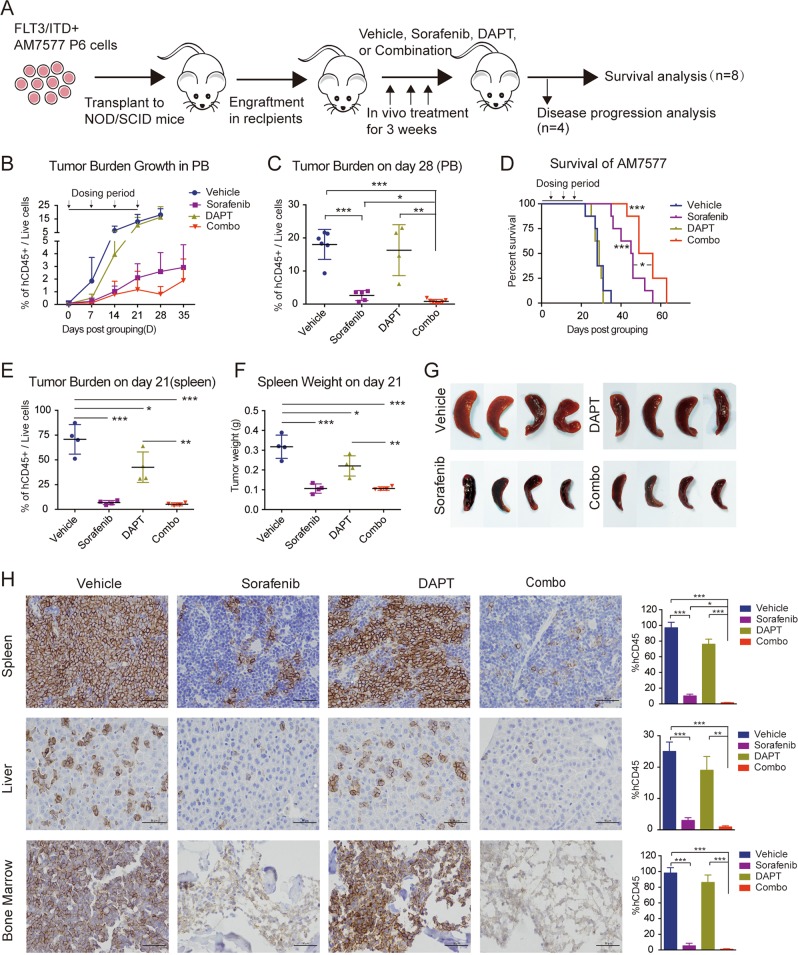
DAPT and sorafenib exhibit therapeutic efficacy in HuKemia^®^ Acute Myelocytic Xenograft Model AM7577 in Female NOD SCID Mice. **a** Schematic diagram of the experimental design. **b** The tumor burden (percentage of human-CD45-positive leukemia cells versus total live cells) growth curves in PB after grouping. Data represent the average ±SD, *n* = 12 mice per cohort. **c** PB engraftment on day 28, represented by the percentage of human CD45^+^ cells. Data represent the average ±SD for cohort vehicle, sorafenib, DAPT, and Combo, *n* = 6, 5, 4, and 7, respectively. (*P *<* 0.05, **P *<* 0.01). **d** Kaplan–Meier survival of the mouse cohorts (*n* = 8 each), indicating the median survival of vehicle (28 days), DAPT (29 days), sorafenib (45.5 days), and Combo (52.5 days) group mice (*P < 0.05, ***P < 0.001). **e–g** Spleen engraftment at the end of treatment, represented by (**e**) the percentage of hCD45^+^ cells, **f** organ weight, and **g** images. Image of the spleen was acquired separately (SONY ILCE-5100), and the original images were trimmed to an equal size of 1.5 cm × 3 cm. Data represent the average of *n* = 4 mice per cohort ±SD (*P < 0.05, **P < 0.01, ***P < 0.001). **h** Representative images of organ infiltration (spleen, liver, bone marrow) analyzed by immunohistochemistry (IHC) staining of human CD45 (hCD45). Statistical analysis is shown on the right. Error bars represent the average of three independent experiments ±SD. (*P < 0.05, **P < 0.01, ***P < 0.001). Images were acquired by NIKON Eclipse ci (Tokyo, Japan) at ×400 magnification. Scale bar, 50 μm.

To investigate the combinatorial effects of FLT3-TKI and GSI in vivo, a PDX model (HuKemia® Acute Myelocytic Xenograft Model AM7577) originating from a FLT3/ITD^+^ patient was used. AM7577 is a well-characterized PDX model with serially transferrable stable AML that faithfully mimics patient characteristics and can be used to test drug efficacy.^22^ After tumor engraftment was detected, the mice were randomly divided into four groups at day 0 and then treated with vehicle, sorafenib, DAPT, or sorafenib plus DAPT (referred as Combo) for 3 weeks (*n* = 12). At the end of administration, four mice from each group were randomly killed for thorough investigation, and the remaining mice (*n* = 8 per cohort) were monitored for survival (Fig. 3a). The mice exhibited good tolerance to individual or combination dosing, with no observed signs of weight loss or other signs of distress (Supplemental Fig. S5). Weekly detection of hCD45 in peripheral blood (PB) by flow cytometry indicated that DAPT monotherapy showed little antitumor effect, while sorafenib monotherapy significantly reduced the tumor burden, and the combination treatment was the most effective (Fig. 3b). Engraftment at day 28 in PB was particularly distinct, with the combined treatment of DAPT plus sorafenib resulting in a significant reduction in leukemic cells compared with sorafenib monotherapy (Fig. 3c). Mice treated with sorafenib had a longer median survival (45.5 days; *P* = 0.0001 vs. vehicle), while DAPT treatment (29 days) led to little difference compared with the vehicle controls (28 days). Overall, mice that received the combined treatment had a statistically longer survival (52.5 days; *P* < 0.0001 vs. DAPT; *P* = 0.0276 vs. sorafenib) (Fig. 3d).

For the mice that were killed in each group, tissues were collected 2 h after the last dose. The combination treatment significantly decreased leukemia infiltration in the spleen, as shown by hCD45 flow cytometry, reduced spleen weight and reduced spleen size (Fig. 3e–g). Strikingly, tumor infiltration of the spleen, liver, and bone marrow, as assessed by immunohistochemical staining of hCD45, was significantly reduced in mice treated with DAPT plus sorafenib compared with the DMSO control or either agent alone (Fig. 3h).

### RNA-seq shows that CXCR3 is involved in synthetic lethality by AC220 and DAPT in FLT3/ITD^+^ cells

**Fig. 4:**
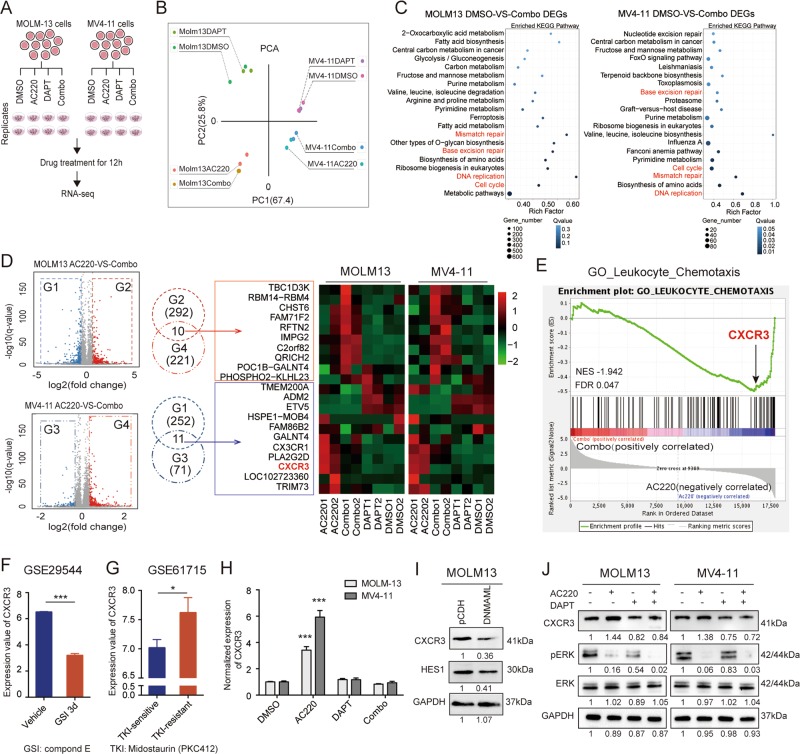
RNA sequencing revealed that CXCR3 plays an important role in the synergistic effects of AC220 and DAPT via reduced ERK activity. MOLM13 and MV4-11 cells were treated with AC220 (2.5 nM) either alone or in combination with DAPT (2.5 µM) for 12 h, and total RNA was extracted from cultured cells for RNA-seq. **a** Schematic view of the study design. **b** Principal component analysis showed that AC220 treatment-induced major changes in the gene expression pattern in both MOLM13 and MV4-11 cell lines. Points represent each sample. The samples in one group are indicated by the same color. **c** The top 20 most enriched pathways identified by KEGG pathway analysis of MOLM13 DMSO-versus-Combo DEGs or MV4-11 DMSO-versus-Combo DEGs. The Q-value is indicated by the scale bar, and the size of the dots reflects the gene number. **d** Genes that shared the same expression pattern of AC220-versus-Combo in both MOLM13 and MV4-11 cell lines were selected (left), and the expression patterns are listed on the right. **e** The plot shows the GSEA of leukocyte chemotaxis genes that were differentially expressed. Data obtained from both MOLM13 and MV4-11 cells were amalgamated for GSEA. The normalized enrichment score (NES) and statistical significance/false-discovery rate Q-value (FDR) are indicated. **f** The publicly available dataset GSE29544 was analyzed to assess the effect of GSI treatment on *CXCR3* expression. CUTLL1 cells were treated with vehicle or GSI for 3 days, and the expression of *CXCR3* is presented (*n* = 3). Student’s *t* test was used for comparisons between two groups. (***P < 0.001). **g** GSE61715 was analyzed to assess the potential role of *CXCR3* in FLT3-TKI resistance. The expression of *CXCR3* in MV4-11 cells that were either sensitive or resistant to PKC412 is shown (*n* = 2). The NCBI GEO2R online tool was used to analyze the dataset. (*P < 0.05). **h** MOLM13 and MV4-11 cells were treated with AC220 (2.5 nM) and/or DAPT (2.5 µM) for 12 h, and the mRNA expression of *CXCR3* was measured in triplicate by qPCR relative to GAPDH expression. Error bars indicate the average fold change vs. DMSO control ±SD. (***P < 0.001). **i** Expression of CXCR3 and HES1 in GFP + MOLM13 cells expressing pCDH or pCDH-DNMAML was determined by western blotting. **j** Expression of CXCR3, phospho-ERK (pERK), total ERK, and GAPDH in MOLM13 and MV4-11 cells was determined by western blotting following the same treatment as described. Images are representative of three independent experiments.

Considering the notable synergistic antitumor effects of combined treatment with FLT3 TKIs and GSIs in FLT3/ITD^+^ cells, we further investigated the mechanisms underlying the observed synthetic lethality. To identify genes involved in the response to combination treatment, we performed RNA-seq using MOLM13 and MV4-11 cells after DMSO, AC220, DAPT, or Combo (AC220 plus DAPT) treatment (Fig. 4a). A summary of the DEGs is shown in Supplemental Fig. S6. Principal component analysis (PCA) showed a separation between MOLM13 and MV4-11 cells as expected, indicating the different genetic backgrounds of these two cell lines. Interestingly, AC220 treatment in both cell lines resulted in clustering of samples by the PCA regardless of DAPT treatment, indicating that AC220 exerts a more profound influence on the transcriptomes of these two cell lines than DAPT (Fig. 4b). In both cell lines, KEGG pathway enrichment analysis of differentially expressed genes (DEGs) between the DMSO and combination treatment demonstrated a significant enrichment for genes involved in pathways including cell cycle, DNA replication, mismatch repair, base excision repair, and cell metabolism (Fig. 4c). Since DAPT elicited limited alterations of the transcriptome profile compared with AC220, we attempted to elucidate the subtle changes introduced by DAPT by comparing DEGs between the AC220 group and the combination group in both cell lines. A total of 21 DEGs with the same expression pattern (10 upregulated in Combo and downregulated in AC220, 11 downregulated in Combo and upregulated in AC220) in both cell lines were selected (Fig. 4d). Among these genes, *CXCR3* was of particular interest because its expression was significantly increased upon AC220 treatment and inhibited by combination treatment. GSEA based on the gene expression profiles of the AC220 and Combo groups in both cell lines showed that the GO term leukocyte chemotaxis was significantly overrepresented in group AC220, in which *CXCR3* was considerably enriched (Fig. 4e). Moreover, in two independent datasets, GSE29544^23^ and GSE61715,^24^ the expression of *CXCR3* was downregulated by a 3-day GSI treatment in the acute lymphocytic leukemia cell line CUTLL1 and upregulated in midostaurin-resistant MV4-11 cells (Fig. 4f, g). Taken together, these results indicated a role for CXCR3 in TKI treatment and the possibility that it could be modulated by GSI.

We verified the gene expression of *CXCR3* by qPCR in MOLM13 and MV4-11 cells after treatment with DMSO, AC220, DAPT, or Combo (AC220 plus DAPT). Consistent with the RNA-seq results, a significant increase in *CXCR3* expression was observed upon treatment with AC220, while cotreatment with DAPT reduced *CXCR3* expression to baseline levels (Fig. 4h). Interestingly, the expression of CXCR3 was significantly decreased in MOLM13 cells expressing DNMAML compared to the control (Fig. 4i), indicating that CXCR3 is a downstream target of Notch signaling. As CXCR3 is a chemokine receptor that has been reported to be associated with ERK phosphorylation, which is also downstream of FLT3 signaling, we evaluated the levels of cellular proteins by western blotting. The results showed that phosphorylation of ERK was strongly inhibited by treatment with AC220 and could be further reduced by combined treatment with AC220 and DAPT (Fig. 4j). The expression of AKT and STAT5 was also measured under the same conditions, and little difference was observed upon DAPT addition (Supplemental Fig. S7).

### The synergistic cytotoxic effects are recapitulated by CXCR3 inhibition and partially rescued by CXCR3 ligand stimulation

**Fig. 5:**
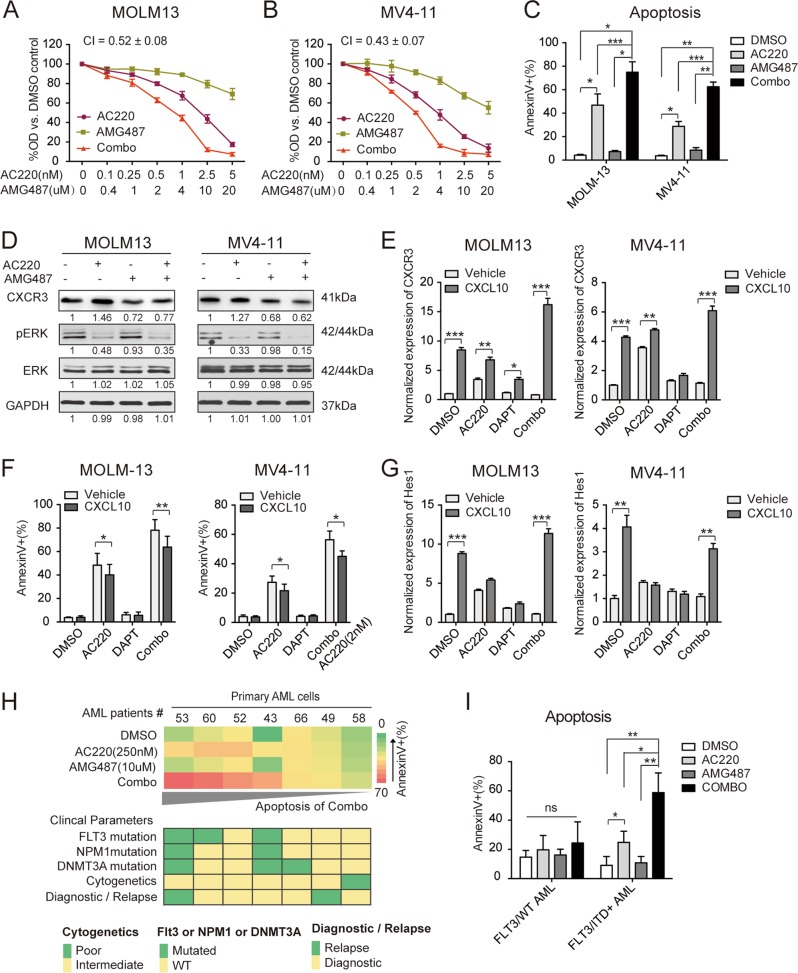
CXCR3 inhibition exerts synergistic cytotoxic effects when combined with AC220, and CXCL10 stimulation partly rescues the apoptosis induced by cotreatment of AC220 and DAPT. **a** MOLM13 and **b** MV4-11 cells were treated with AC220 (0–5 nM) either alone or in combination with AMG487 (0–25 µM) for 72 h, and cell proliferation was measured in triplicate using the CCK-8 assay. **c** Annexin V binding was assessed in MOLM13 and MV4-11 cells treated with AC220 (2.5 nM) and/or AMG487 (10 µM) in triplicate for 48 h. Data represent the average of three independent experiments ± SD. (*P *<* 0.05; **P *<* 0.01, ***P *<* 0.001). **d** MOLM13 and MV4-11 cells were treated with AC220 (2.5 nM) either alone or in combination with AMG487 (10 µM) for 12 h, and expression levels of CXCR3, phospho-ERK (pERK), total ERK, and GAPDH were measured by western blotting of cellular proteins. Images are representative of three independent experiments. MOLM13 and MV4-11 cells were treated with DMSO, AC220 (2.5 nM), DAPT (25 µM), or Combo (AC220 combined with DAPT), in addition to either vehicle or CXCL10 (10 ng/ml). **e** The expression of *CXCR3* at 12 h was measured in triplicate by qPCR relative to *GAPDH* expression. **f** Apoptosis at 48 h was measured in triplicate by Annexin V staining. **g** Expression of *Hes1* at 12 h was measured in triplicate by qPCR relative to *GAPDH* expression. Data represent the average of three independent experiments ±SD. (*P < 0.05, **P *<* 0.01, ***P < 0.001). **h** Primary AML samples (*n* = 7) were treated with AC220 (250 nM) and/or AMG487 (10 μM) for 48 h. **i** Apoptosis at 48 h was measured in triplicate by Annexin V staining. The corresponding clinical parameters for each AML patient are presented below. Samples were ranked by their apoptosis rate under the combined treatment. Statistical analysis of apoptosis is presented on the left. FLT3/ITD^+^ (*n* = 3) and FLT3/WT (*n* = 4). Data represent the average of three independent experiments ±SD. (*P < 0.05, **P *<* 0.01).

To further investigate the role of CXCR3 modulation in the synergistic effects observed with combined AC220 and DAPT treatment, AMG487, an antagonist that blocks the binding of the ligand CXCL10 or CXCL11 to CXCR3, was used. Treatment of MOLM13 and MV4-11 cells with AMG487 in combination with AC220 mimicked the effects of DAPT combined with AC220, with synergistic effects observed on both proliferation and apoptosis (Fig. 5a–c, Supplemental Fig. S9). Upon AMG487 treatment, the CXCR3 protein levels in MOLM13 and MV4-11 cells were significantly decreased, and the upregulation of CXCR3 by AC220 was also reduced compared to controls. Compared that induced by AC220 treatment alone, the expression of phospho-ERK was further decreased following cotreatment with AMG487, consistent with the modulation pattern observed in combination with DAPT (Fig. 5d).

In contrast, CXCR3 was overexpressed when cells were stimulated by its ligand CXCL10, which protected MOLM13 and MV4-11 cells from AC220 or combination (AC220 plus DAPT) treatment-induced apoptosis (Fig. 5e, f, Supplemental Fig. S9). Interestingly, *Hes1* upregulation by CXCL10 stimulation was observed in MOLM13 and MV4-11 cells treated with DMSO or the combination of AC220 and DAPT (Fig. 5g), indicating crosstalk between Notch signaling and CXCR3.

To preliminarily explore the therapeutic potential of combinational inhibition of CXCR3 and FLT3 in AML, we examined the effects of AC220 combined with AMG487 on primary AML cells. Mononuclear cells from peripheral blood samples of 7 AML patients were treated with AC220 (250 nM) and/or AMG487 (10 µM) for 48 h. As shown in Fig. 5h, increased apoptosis in cells treated with both AC220 and AMG487 was more likely to be observed in specimens from FLT3/ITD^+^ patients. Statistically, a significant increase in Annexin V+ binding was detected following combination treatment with AMG487 and AC220 in FLT3/ITD^+^ primary samples compared to either AMG487 or AC220 alone (Fig. 5i, Supplemental Fig. S9). On the other hand, AMG487 treatment (0–25 µM) alone did not affect apoptosis of peripheral blood mononuclear cells from healthy donors (Supplemental Fig. S8).

## Discussion

In the present study, we examined the combinational effects of TKI and GSI on FLT3/ITD + AML in vitro and in vivo. The synergistic antitumor efficacy was confirmed in primary samples, FLT3/ITD+ cell lines and xenograft models. Using transcriptome profiling, we identified a chemokine receptor, CXCR3, which was significantly upregulated in response to TKI exposure and dramatically downregulated upon exposure to GSI and TKI together. Further analysis with CXCR3 inhibitor and ligand suggested that CXCR3 was partially responsible for the observed synergy by modulating ERK signaling.

In hematopoietic malignancies, the role of Notch signaling is highly cell context specific. Notch has been identified as an oncogene in T-acute lymphoblastic leukemia, chronic lymphocytic leukemia, B cell leukemias, and lymphomas.^10^ Notch signaling promotes multiple myeloma cell proliferation, whereas Dll1/Notch activation contributes to bortezomib resistance.^25^ In chronic myelomonocytic leukemia, a tumor suppressor role for the Notch pathway has been reported, supporting a loss-of-function hypothesis.^26^ However, the role of Notch signaling in AML remains controversial due to its relatively low expression and rare activation in human AML.^19^ In conflicting studies, Notch signaling has been shown to promote self-renewal, induce/inhibit differentiation to monocytes, and induce apoptosis in myeloid precursors.^27–29^ Reactivation of the Notch pathway has been reported to induce differentiation, growth arrest, and apoptosis both in primary human AML samples and in mouse models.^19^ However, another group reported a full range of responses, from proliferation to growth arrest, following exposure of AML patient samples to Notch ligand.^30^ A possible explanation for these controversial findings is that AML is a highly heterogeneous disease and must be analyzed with respect to the specific genetic background. We sought to determine whether Notch signaling is modulated following treatment with TKIs in FLT3/ITD + AML. Curiously, the expression of several Notch-related genes, including Hes1, a known downstream target of Notch signaling, was increased after TKI exposure in both FLT3/ITD + AML primary samples and cell lines. Notably, Takayasu Kato et al.^31^ reported that Hes1 binds directly to the promoter region of the FLT3 gene and downregulates promoter activity. It has also been reported that the Hes1 protein directly binds to STAT3 and promotes STAT3 phosphorylation and activation.^32^ Thus, it is possible that via induction of Hes1, FLT3/ITD + AML cells could become desensitized to TKI and reduce its effect on FLT3 signaling by blocking FLT3 expression and resulting in a shift to alternative signaling pathways. We tested the hypothesis that inhibition of Notch signaling increases the sensitivity of FLT3/ITD + AML cells to TKIs, although the exact mechanisms that lead to the activation of Notch signaling upon TKI treatment require further investigation. Consistent with previous reports that combine Notch inhibition with anti-leukemic drugs in myeloma,^33^ B-ALL^34,35^, and CLL,^36^ our research supports the idea of dual inhibition of FLT3 and NOTCH signaling with small molecules as a potential strategy for FLT3/ITD + AML treatment.

**Fig. 6:**
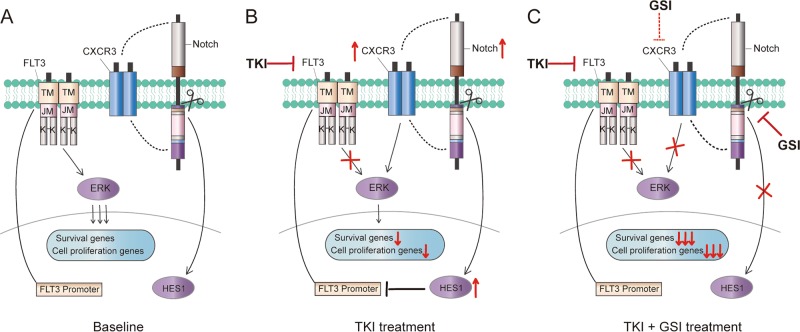
Proposed model showing the mechanism of combined FLT3 and Notch inhibition. **a** At baseline, FLT3-ITD signaling results in high ERK activity and, therefore, high expression of survival and cell proliferation genes. **b** Following treatment with FLT3-TKIs, ERK activity mediated by FLT3-ITD signaling is inhibited, while Notch signaling and CXCR3 expression are upregulated, resulting in alternative ERK activation. **c** GSI addition abrogates the rebound by blocking Notch signaling and decreasing CXCR3 activity, thus resulting in extreme repression of ERK activity and a synergistic antitumor effect.

Thus far, several mechanisms have been proposed to explain TKI treatment failure, including the acquisition of secondary mutations of the targeted kinase and the activation of alternative pathways.^37,38^ Based on studies on the molecular mechanisms underlying the observed synergy, we suspect that CXCR3 could be a possible escape route that bypasses FLT3 signaling. CXCR3, a member of the G protein-coupled receptors, is expressed on the surface of a plethora of cell types, including monocytes, lymphocytes, natural killer, endothelial, and cancer cells.^39^ Upon binding to four major chemokine ligands, CXCL4, CXCL9, CXCL10, and CXCL11, CXCR3 exhibits various functions in tumor cell proliferation, cancer metastasis, and inflammatory diseases.^39,40^ Although the importance of CXCR3 in AML is not fully understood, Manuel Ramírez^41^ reported that CXCL10/CXCR3 signaling plays an important role in the relapse of childhood acute lymphoblastic leukemia by inducing chemotaxis and diminishing chemotherapy-related apoptosis. In line with previous reports showing that the CXCL10/CXCR3 axis activates MEK and ERK signaling in several cancer types,^42–44^ our results demonstrated that ERK signaling was modulated by TKI-induced expression of CXCR3, which could be abrogated by GSI or CXCR3-specific inhibitors in FLT3/ITD + AML cells. This phenomenon is consistent with the observation that ERK signaling can be reactivated in FLT3/ITD + AML cells treated with TKIs,^8^ and it supports the notion that CXCR3 is, at least partially, responsible for the TKI-induced changes in signaling (Fig. 6a–c). Notably, another chemokine receptor, CXCR4, has been reported to be stimulated by FLT3 mutation and contributes to the resistance of AML cells to FLT3 inhibitors under stromal coculture conditions. It is possible that the microenvironment is also involved in CXCR3 signaling to provide the chemokine needed.^45^ Moreover, the ability of both a GSI and a CXCR3 inhibitor to block CXCR3 signaling raises the question of how NOTCH signaling modulates the CXCL10/CXCR3/ERK axis. Considering that the toxicity caused by GSI in humans may not be well tolerated, it is of interest whether CXCR3 inhibitor is preferable to GSI in combination with TKI against FLT3/ITD + AML for safety concerns.

Overall, this study provides new insights into the role of NOTCH signaling in response to TKI treatment and highlights CXCR3/ERK signaling as an alternative pathway of FLT3 inhibition, suggesting a combinational approach of TKI and GSI to treat FLT3/ITD^+^ AML.

## Methods

### Cell lines and cell culture

The human AML cell lines MOLM13, THP-1, OCI-AML3, and SKM-1 were obtained from ATCC (American Type Culture Collection) in 2011. The MV4-11 cell line was purchased from DSMZ (Deutsche Sammlung von Mikroorganismen und Zelkulturen, Braunschweig, Germany) in 2011. All human cell lines were authenticated by short tandem repeat DNA profiling at CCTCC (China Center for Type Culture Collection). The SKM-1 FLT3-ITD knock-in mutant cell lines were generated using the CRISPR/Cas9 system according to the reported method.^46,47^ All cell lines except MV4-11 and OCI-AML3 were cultured in RPMI 1640 medium (Gibco, Waltham, MA); MV4-11 and OCI-AML3 cells were maintained in Iscove’s modified Dulbecco’s medium (Gibco) and a-Minimum Essential Medium (Gibco), respectively. All media contained 10% fetal bovine serum (Gibco). Cells were maintained in a 37 °C humidified atmosphere containing 5% CO_2_.

### Proliferation assay

For proliferation assays, cells were seeded in a 96-well plate at 40,000 cells/100 µL per well. Inhibitors were added, and the cells were incubated at 37 °C for 72 h. Cell viability was measured using a Cell Counting Kit-8 (CCK-8, Dojindo, Kumamoto, Japan) according to the manufacturer’s instructions. Each experiment was performed in triplicate. Combinatorial index (CI) values across a range of drug concentrations were calculated using CalcuSyn software (Biosoft, Cambridge, UK) with the Chou-Talalay equation.^48^

### Apoptosis analysis

Cells were seeded at a density of 2 × 10^5^ cells/mL in the presence or absence of compounds for 48 h. Harvested cells were washed and resuspended in binding buffer containing Annexin V-FITC and propidium iodide (BD Biosciences, Bedford, MA). After incubation for 15 min, the samples were subjected to FACS analysis using a Beckman Coulter FC500 flow cytometer (Beckman Coulter, Pasadena, CA).

### Colony formation assay

Cells were suspended in culture medium containing 1.6% methylcellulose (Sigma-Aldrich, St Louis, MO) and 20% FBS (Gibco by Thermo Fisher Scientific, Waltham, MA) and seeded in a 24-well plate at 100–200 cells per well. Colonies were counted 14 days after plating, and each measurement was performed in triplicate.

### Human samples

Human peripheral blood (PB) samples from AML patients were collected under a protocol that was approved by the Institutional Review Board of Tongji Medical College, Huazhong University of Science and Technology (permit number 2018-S385), with patients informed consent acquired in accordance with the Declaration of Helsinki. The main clinical characteristics are shown in Supplemental Table S1. Mononuclear cells were isolated by density-gradient centrifugation (Ficoll-Paque, Solarbio, Beijing, China) and cultured in RPMI 1640 (Gibco) supplemented with 10% FBS for drug treatments. Cells were then subjected to apoptosis analysis, proliferation analysis, and flow cytometry analysis.

### In vivo study

Animal experiments were conducted in a specific pathogen-free facility at Crown Bioscience (Nanjing, China). All protocols were approved by the Institutional Animal Care and Use Committee (IACUC) of Crown Bioscience (Project number: E3433-T1702). Five- to 6-week-old NOD/SCID female mice were obtained from Beijing Anikeeper Bio-Technology. Each mouse was injected with 1.2 × 10^6^ AM7577 cells (passage 6) via intravenous injection after sublethal irradiation. Peripheral blood was collected retro-orbitally each week, and engraftment was assessed by flow cytometric measurements of human-CD45. When the average tumor burden reached 0.5–2% of human-CD45+ cells in PB, mice were allocated into four groups (12 mice in each group) by stratified randomization based on body weight. After grouping, mice were treated with vehicle, sorafenib (3 mg/kg, QD, *p.o*.), DAPT (10 mg/kg, QOD, *i.p*.), or combo (3 mg/kg sorafenib plus 10 mg/kg DAPT) for 3 weeks. On the day of the last dosing, four mice in each group were killed randomly. Bone marrow, liver, and spleen were harvested for thorough analysis including gross examinations, flow cytometric measurements, and immunohistochemistry analysis under protocols described previously.^22^ The other mice continued to be bred and analyzed for human-CD45 weekly until they reached the survival end point. Lab scientists responsible for drug administration and assessing the mouse status were blinded to the experimental conditions and detailed study design.

### RNA-seq analysis

For RNA-seq analysis, MV4-11 and MOLM13 cells were treated in duplicate with DMSO, 2.5 nM AC220, 25 µM DAPT, or combo (2.5 nM AC220 plus 25 µM DAPT) for 12 h. A total of 16 samples were sequenced using the BGISEQ-500 platform. Differentially expressed genes (DEGs) were identified using the DESeq package. Genes showing altered expression with a false-discovery rate *P* < 0.05 and more than 1.5-fold changes were considered differentially expressed. For details on data analysis, see Supplemental Methods.

### Statistical analysis

The results are presented as the mean ± SD. Student’s *t* test was used for comparisons between two groups. For multiple comparisons, ordinary one-way ANOVA with Tukey’s multiple comparison test was used. For survival analysis, Kaplan–Meier survival curves were generated, and the log-rank test was performed. All data were analyzed using GraphPad Prism 6.0c (GraphPad Software, La Jolla, CA), and *P* < 0.05 was considered statistically significant.

## Supplementary information

read-me for supplementary information

Author List Changes Approval form

Supplementary Information

## Data Availability

Detailed information on the reagents and primers may be found in Supplemental Tables S2 and S3. RNA-seq data are available at GEO under accession number GSE126933. For original data, please contact jeff_wangjue@hotmail.com.
