# Supplementary material for: Combined inhibition of Notch and FLT3 produces synergistic cytotoxic effects in FLT3/ITD+ acute myeloid leukemia
Source: Signal Transduct Target Ther. 2020 Mar 13;5:21. doi: 10.1038/s41392-020-0108-z (PMC7067872; doi:10.1038/s41392-020-0108-z)
Supplement: Supplementary file 1 — read-me for supplementary information [file 41392_2020_108_MOESM1_ESM.docx]

The supplementary information contains three parts.

Part 1: supplemental methods, including detailed methods of RNA isolation and quantitative PCR, western blot analysis, flow cytometry analysis, generation of FLT3/ITD knock-in cell lines, cell transfection and RNA-seq analysis and gene set enrichment analysis (GSEA).

Part 2: supplementary figures:

- Supplemental Figure 1. Expression of active forms of Notch
- Supplemental Figure 2. Effect of AC220 and DAPT on FLT3/WT cells and combinatorial effect of FLT3 TKIs and GSIs on FLT3/ITD+ cells
- Supplemental Figure 3. Generation and verification of CRISPR/Cas9-mediated FLT3/ITD mutation knock-in in SKM-1 cells
- Supplemental Figure 4. AC220 combined with DAPT has little effect on normal CD34+ stem/progenitor cells and DAPT alone does not affect apoptosis of peripheral blood mononuclear cells from healthy donors
- Supplemental Figure 5. Treatment with sorafenib and DAPT alone or in combination is well tolerated
- Supplemental Figure 6. Summary of differentially expressed genes (DEGs)
- Supplemental Figure 7. Expression downstream of FLT3
- Supplemental Figure 8. AMG487 had little effect on peripheral blood mononuclear cells from healthy donors
- Supplemental Figure 9. Representative raw plots for apoptosis assay.

Part 3: Supplementary Tables:

- Supplemental Table 1. AML patient characteristics
- Supplemental Table 2. Information on the oligonucleotides used in this paper
- Supplemental Table 3. Information on the reagents used in this paper
