# Supplementary figures and images for: Combined inhibition of Notch and FLT3 produces synergistic cytotoxic effects in FLT3/ITD+ acute myeloid leukemia
Source: Signal Transduct Target Ther. 2020 Mar 13;5:21. doi: 10.1038/s41392-020-0108-z (PMC7067872; doi:10.1038/s41392-020-0108-z)

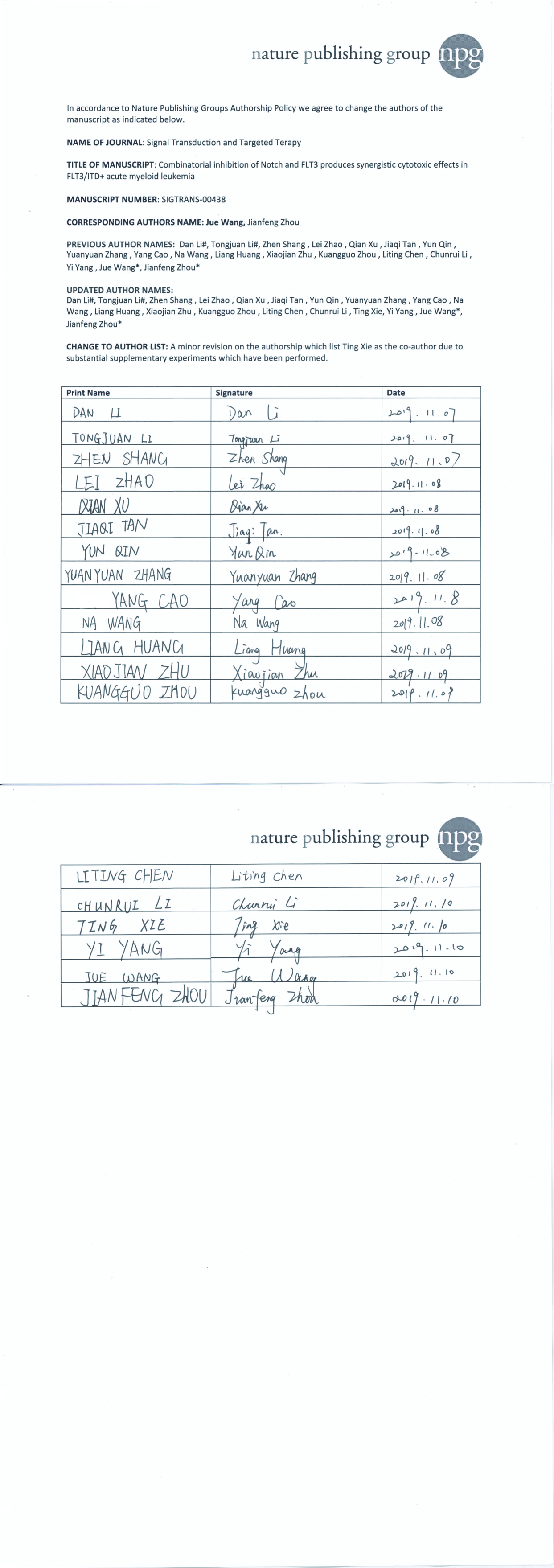

Supplement: Supplementary file 2 — Author List Changes Approval form [file 41392_2020_108_MOESM2_ESM.jpg]
