# Supplementary material for: Combined inhibition of Notch and FLT3 produces synergistic cytotoxic effects in FLT3/ITD+ acute myeloid leukemia
Source: Signal Transduct Target Ther. 2020 Mar 13;5:21. doi: 10.1038/s41392-020-0108-z (PMC7067872; doi:10.1038/s41392-020-0108-z)
Supplement: Supplementary file 3 — Supplementary Information [file 41392_2020_108_MOESM3_ESM.pdf]

Supplementary Information

Combinatorial inhibition of Notch and FLT3 produces synergistic cytotoxic effects in FLT3/ITD$^+$ acute myeloid leukemia

Supplementary Methods

RNA isolation and quantitative PCR

After the designated treatments, cells were collected using TRIzol reagent (Invitrogen, Thermo Fisher), and total RNA was extracted from cultured AML cells using Direct-zol RNA kits (Zymo Research, Beijing, China) according to the manufacturer's instructions. Complementary DNA (cDNA) was synthesized using a TaKaRa RNA PCR Kit (Takara, Otsu, Japan). Quantitative RT-PCR reactions were carried out on a CFX96 Touch^{TM} Real-Time PCR Detection System (Bio-Rad, CA, USA). Relative mRNA expression was quantified according to the comparative Ct method with normalization to *GAPDH*. The primers used are displayed in **Supplementary Table2**.

Western blot analysis

Exponentially growing cells were plated in culture medium supplemented with inhibitors at the indicated concentration. After incubation for 12 h, the cells were harvested, washed, and then lysed in RIPA buffer (ServeBio, Wuhan, China)

supplemented with protease inhibitors (ServeBio). The lysates were quantified by the Bradford assay, and equal amounts of lysates were separated by SDS-PAGE and transferred to PVDF membranes (GE Healthcare, NJ, USA). Proteins were detected by immunoblotting using ChemiDoc XRS+ imaging System (Bio-Rad) following incubation with primary antibodies and appropriate secondary antibodies. Images were obtained and analyzed using Image Lab Software (Bio-Rad). The antibodies used are listed in **Supplementary Table 3**.

Flow cytometry analysis

Flow cytometry analysis was performed using BD FACSCalibur machine (BD Biosciences) or Beckman Coulter FC500 flow cytometer (Beckman Coulter). To measure the CD34+ cell proportion of human peripheral blood samples, mononuclear cells were collected after isolation and the designated drug treatments. Cells were then washed two times with PBS and stained with anti-CD34 antibody (Biolegend, CA, USA) for 30 minutes at 4℃ After washing, the samples were subjected to flow cytometry analysis.

To quantify leukemic engraftment in the orthotopic AML xenograft model, the PB and spleen cells were isolated after red cell lysis, labeled with anti-human CD45 antibody (Biolegend) and analyzed by flow cytometry. Data were analyzed using Cytoexpert software (Beckman Coulter) and FlowJo software (version 10.0.7r2; TreeStar, USA).

Generation of FLT3/ITD knock-in Cell Lines

FLT3/ITD knock-in cell lines were generated according to the reported method [1]. To generate short guide RNA (sgRNA) constructs, sequences targeting the 14th exon of *FLT3* were loaded at crispr.mit.edu. The BbsI restriction site (CACC) was added to the selected oligos. The oligomers were then annealed and cloned into pSpCas9(BB)-2A-GFP (PX458) under control of the U6 promoter (Addgene # 48138).

To construct the FLT3/ITD donor vector, an up homologous arm located upstream of the FLT3/ITD mutation site and a down homologous arm located downstream of the FLT3/ITD mutation site, approximately 800bp in length on each side, were amplificated by PCR (KOD Fx, Toyobo, Osaka, Japan). Genomic DNA from the primary blast of a FLT3/ITD+ patient was used as a template. The Loxp-BSD-Loxp locus was inserted as a selective marker. Donor segments were then cloned into the vector using pEASY–simple T1 Cloning Kit (TansGene Biotech, Beijing, China) according to the instructions. To avoid unexpected cutting, a synonymous mutation in the donor vector was constructed using the QuikChange® Lightning Site-Directed Mutagenesis Kit (Stratagene, Agilent, CA, USA) following the manufacturer's instructions.

SKM-1 cells (1×10^6 cells per well in 1 mL culture medium in a 12-well plate) were co-transfected with 1 μg sgRNA plasmid and 2 μg donor plasmid using Lonza

4D nucleofector (Lonza, Basel, Switzerland) according to a previously reported method [2]. GFP+ cells were sorted by FACS after 2 weeks of BSD drug selection and seeded in a 96-well plate, with a single cell per well. Subsequently, the cells were cultured, expanded and genotyped. For genotyping, PCR reactions were performed using KOD Fx Polymerase (Toyobo) following the manufacturer's instructions. The PCR products were gel-extracted, and the insertion was confirmed by DNA sequencing. FLT3-ITD-Loxp-BSD-Loxp knock-in cells were transfected with 1 μg pBS505 EF1 alpha EGFP Cre (Addgene #11955), and GFP+ cells were sorted by FACS after 72 h and seeded at a single cell per well. After cell expansion, genotyping was performed again, and SKM-1 FLT3-ITD knock-in cells were finally identified. The primers used for genotyping are listed in **Supplementary Table 2**. Conversely, the FLT3/ITD knock-in clones were validated by q-PCR and immunoblotting.

Cell transfection

For *Hes1* knockdown, equal amount of non-silencing (NS) or Hes1-targeted (Hes1_1, Hes1_2 and Hes1_3) siRNAs were transfected using Lonza 4D nucleofector (Lonza, Basel, Switzerland) according to the manufacturer's protocol. AC220 was added 24h after transduction. Lentivirus expressing pCDH vector and DNMAML were kindly provided by Hudan Liu (Medical Research Institute, Wuhan University, Wuhan, China) and lentiviral transduction was performed as described before [3]. For transfection, $2x10^5$ exponentially growing cells were seeded in 0.5mL culture medium

with additional 4μg/mL polybrene (Genechem). Lentivirus were added at an MOI of 25. Three days after transduction, MOLM13 cells were selected in culture medium containing 1ug/mL puromycin for 1 week.

RNA-seq analysis and Gene Set Enrichment Analysis (GSEA)

Principal component analysis (PCA) was performed with R package gmodels (http://www.r-project.org/). The biological pathways were identified from Kyoto Encyclopedia of Genes and Genomes (KEGG, http://www.genome.jp/kegg/). GSEA was performed by comparison of normalized gene expression data obtained from the AC220 and combo groups. Genes were compared to gene set collections from the molecular signatures database (MsigDB) using the online portal (http://software.broadinstitute.org/gsea/ msigdb/index.jsp).

Supplemental References

1. Ran FA, et al. Genome engineering using the CRISPR-Cas9 system. *Nat Protoc*. 2013;8(11):2281-308.

2. Wang J, et al. TALENs-mediated gene disruption of FLT3 in leukemia cells: Using genome-editing approach for exploring the molecular basis of gene abnormality. *Sci Rep*. 2015;5(1).

3. Ye Q,et al. Small molecule activation of NOTCH signaling inhibits acute myeloid leukemia. *Sci Rep* 2016; 6: 26510.

Supplementary Figures:

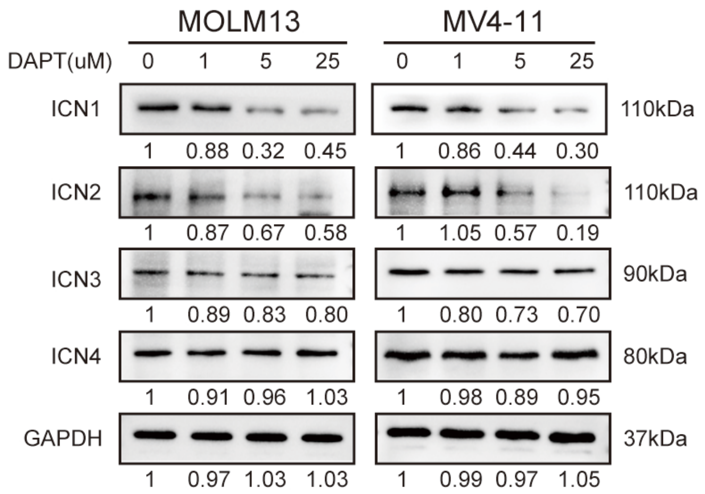

Supplemental Figure 1. Expression of active forms of Notch

The expression of ICN1, ICN2, ICN3 and ICN4 in Molm13 and MV4-11 cells were measured by western blotting following treatment with indicating concentrations of DAPT for 12 h. GAPDH was used as a loading control. Images are representative of three independent experiments.

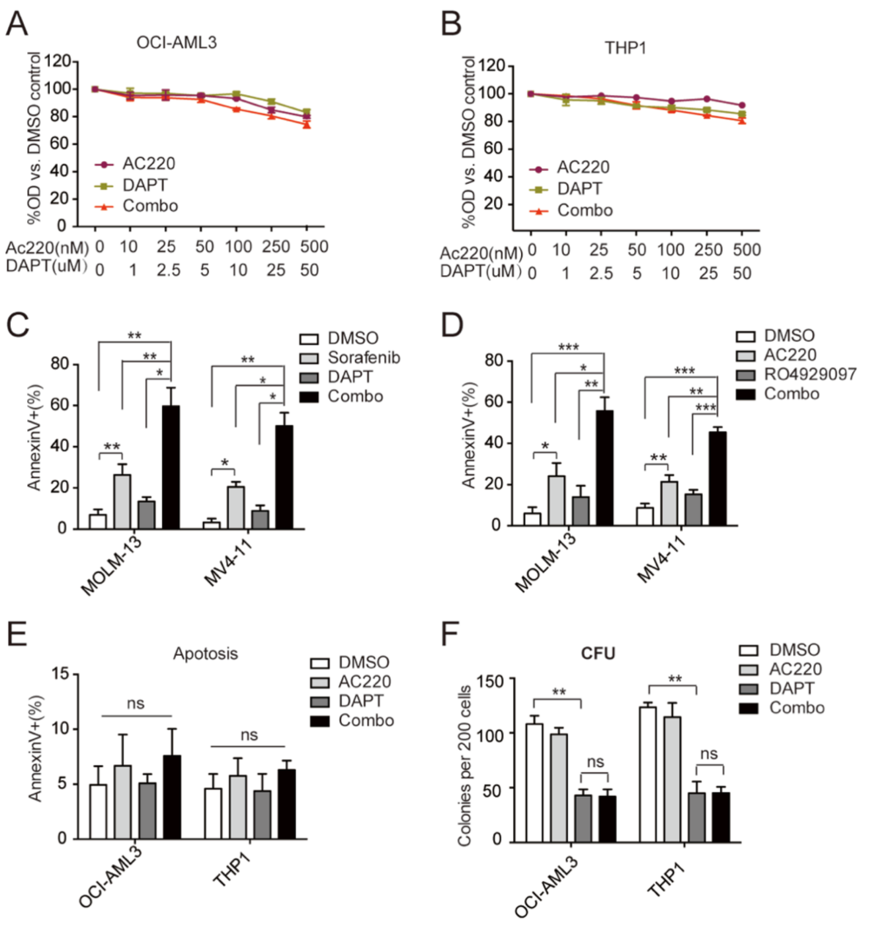

Supplemental Figure 2. Effect of AC220 and DAPT on FLT3/WT cells and combinatorial effect of FLT3 TKIs and GSIs on FLT3/ITD+ cells

FLT3/WT AML cells, **(A)** OCI-AML3 and **(B)**THP1 cells were treated with AC220 (0-500 nM) either alone or in combination with DAPT (0-50 μM) for 72 h, and cell proliferation was measured in triplicate by the CCK-8 assay. Error bars indicate the average % OD ± SD. MOLM13 and MV4-11 cells were treated with **(C)** sorafenib (20 nM) alone or in combination with DAPT (25 μM) or **(D)** AC220 (2.5 nM) alone or in combination with RO4929097 (25 μM). Apoptosis was measured by Annexin V binding at 48 h; data represent the average of three independent experiments ± SD. (*, $P < 0.05$, **, $P < 0.01$; ***, $P < 0.001$). **(E)** OCI-AML3 and THP1 cells were treated with AC220 (250 nM) and/or DAPT (25 μM) for 48 h, and apoptosis was measured in triplicate by Annexin V binding. Error bars indicate the average of three independent experiments ± SD. (ns, not significant). **(F)** CFU counts after 14 days of plating of 2×10^2 OCI-AML3 or THP-1 cells treated in triplicate with AC220 (100 nM) and/or DAPT (10 μM). Data indicate the average colony number ± SD (**, $P < 0.01$).

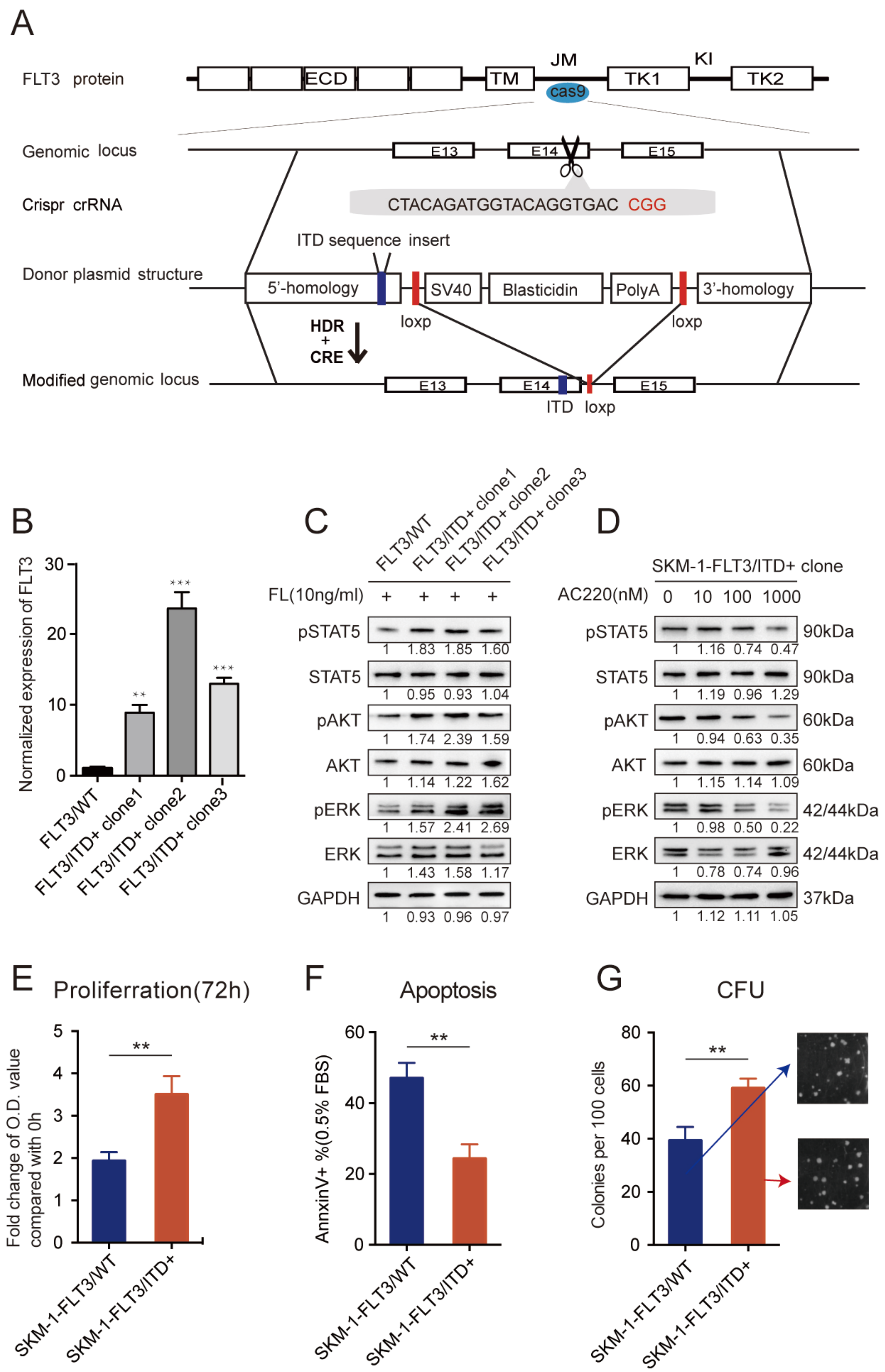

A
FLT3 protein
ECD
TM
JM
cas9
TK1
KI
TK2
Genomic locus
E13
E14
E15
Crispr crRNA
CTACAGATGGTACAGGTGAC CGG
ITD sequence insert
Donor plasmid structure
5'-homology
SV40
Blasticidin
PolyA
3'-homology
loxp
loxp
HDR + CRE
Modified genomic locus
E13
E14
E15
ITD loxp
B
Normalized expression of FLT3
FLT3/WT
FLT3/ITD+ clone1
FLT3/ITD+ clone2
FLT3/ITD+ clone3
C
FL(10ng/ml) + + + +
pSTAT5 1 1.83 1.85 1.60
STAT5 1 0.95 0.93 1.04
pAKT 1 1.74 2.39 1.59
AKT 1 1.14 1.22 1.62
pERK 1 1.57 2.41 2.69
ERK 1 1.43 1.58 1.17
GAPDH 1 0.93 0.96 0.97
D
SKM-1-FLT3/ITD+ clone
AC220(nM) 0 10 100 1000
pSTAT5 1 1.16 0.74 0.47 90kDa
STAT5 1 1.19 0.96 1.29 90kDa
pAKT 1 0.94 0.63 0.35 60kDa
AKT 1 1.15 1.14 1.09 60kDa
pERK 1 0.98 0.50 0.22 42/44kDa
ERK 1 0.78 0.74 0.96 42/44kDa
GAPDH 1 1.12 1.11 1.05 37kDa
E
Proliferration(72h)
Fold change of O.D. value compared with 0h
SKM-1-FLT3/WT
SKM-1-FLT3/ITD+
F
Apoptosis
AnnxinV+ %(0.5% FBS)
SKM-1-FLT3/WT
SKM-1-FLT3/ITD+
G
CFU
Colonies per 100 cells
SKM-1-FLT3/WT
SKM-1-FLT3/ITD+

Supplemental Figure 3. Generation and verification of CRISPR/Cas9-mediated FLT3/ITD mutation knock-in in SKM-1 cells

(A) Schematic overview of the strategy used to knock-in the FLT3/ITD mutation in FLT3 exon 14. **(B)** Quantitative RT-PCR analysis of *FLT3* transcript levels in SKM-1 wild type cells (FLT3/WT) and three FLT3/ITD knock-in clones relative to *GAPDH*. Error bars indicate the average fold change relate to FLT3/WT cells of three independent experiments ± SD (**$P < 0.01$; ***, $P < 0.001$). **(C)** Representative image of immunoblot analysis of the signaling mediators downstream of FLT3 in SKM-1 wild type and FLT3/ITD+ clones. The cells were pre-starved of growth factors for 12 h and subsequently exposed to FLT3 ligand (0.1ng/ml) for 10 minutes before harvesting. **(D)** Expression of FLT3 signaling downstream mediators in a single FLT3/ITD+ clone named SKM-1-1D5 by western blotting following treatment with AC220 (0-1000 nM). Images are representative of three independent experiments. **(E)** SKM-1-WT and SKM-1-1D5 cells were plated, and proliferation at 72 h was measured in triplicate by the CCK-8 assay. Data represent the average of three independent experiments ± SD (**, $P < 0.01$). **(F)** SKM-1-WT and SKM-1-1D5 cells were cultured in 1640 medium containing 0.5% FBS, and apoptosis at 96 h was measured in triplicate by Annexin V binding. Data represent the average of three independent experiments ± SD. (*, $P < 0.05$, **, $P < 0.01$; ***, $P < 0.001$). **(G)** The colony-forming assay was performed in methylcellulose based culture medium after 14 days of plating of 100 SKM-1-WT or SKM-1-1D5 cells per well. Quantitative analysis of the colony numbers was shown in the plot on the left. Representative images are shown on the right. Scale bar, 20 mm. Error bars represent the average of three independent experiments ± SD. (**$P < 0.01$)

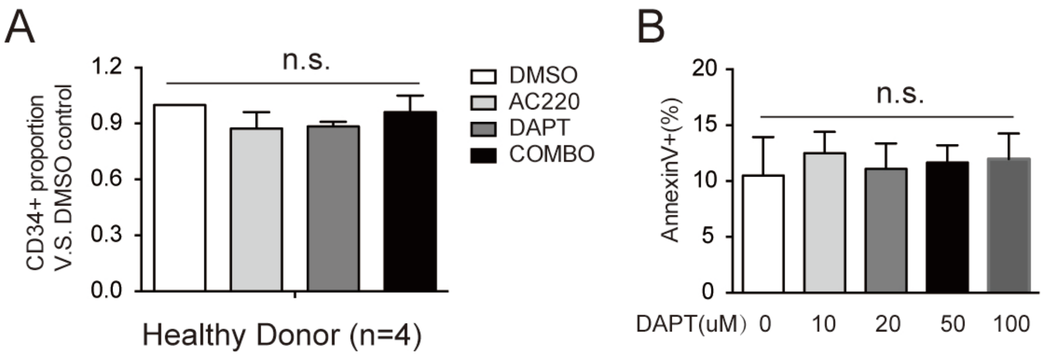

Supplemental Figure 4. AC220 combined with DAPT has little effect on normal CD34+ stem/progenitor cells and DAPT alone does not affect apoptosis of peripheral blood mononuclear cells from healthy donors

(A) Normal peripheral blood samples obtained from healthy donors (n=4) were treated with AC220 (250 nM) either alone or in combination with DAPT (25 μM), and CD34 expression was measured by flow cytometry at 48 h. **(B)** Peripheral blood mononuclear cells obtained from healthy donors(n=4) were treated with 0~100 μM DAPT for 48 hours and apoptosis was measured by Annexin V binding. Error bars indicate the average ± SD (ns. not significant).

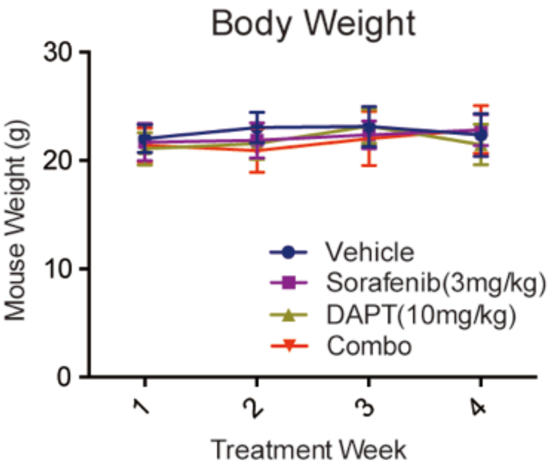

Supplemental Figure 5. Treatment with sorafenib and DAPT alone or in combination is well tolerated

The mouse weight for each cohort, measured once a week, is shown (n=12). Error bars indicate the average ± SD.

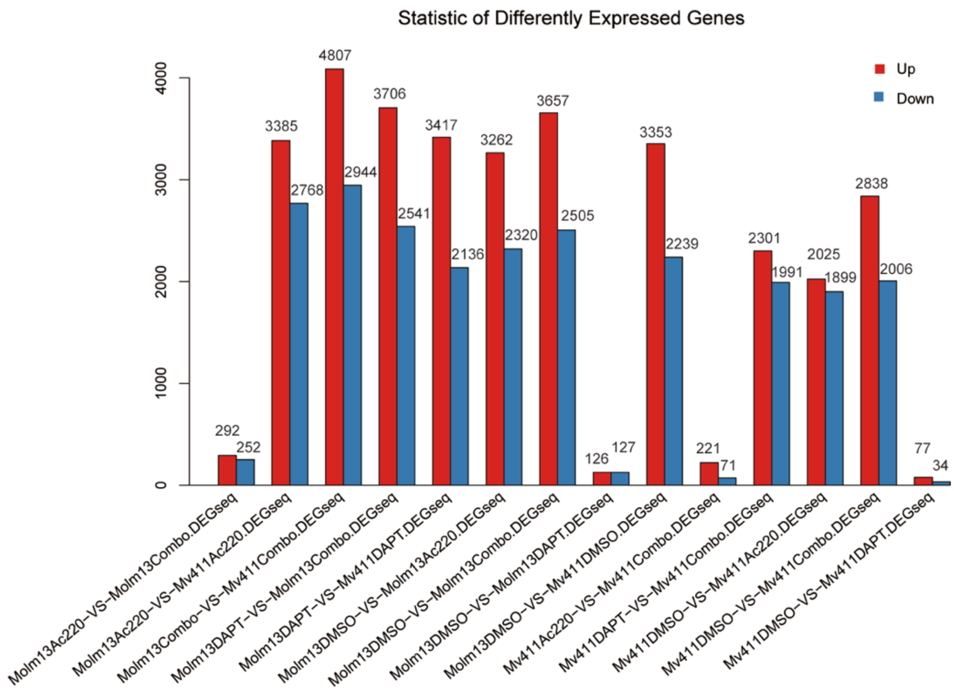

Supplemental Figure 6. Summary of differentially expressed genes (DEGs)

The numbers of differentially expressed genes (DEGs) identified by the gene expression level are presented.

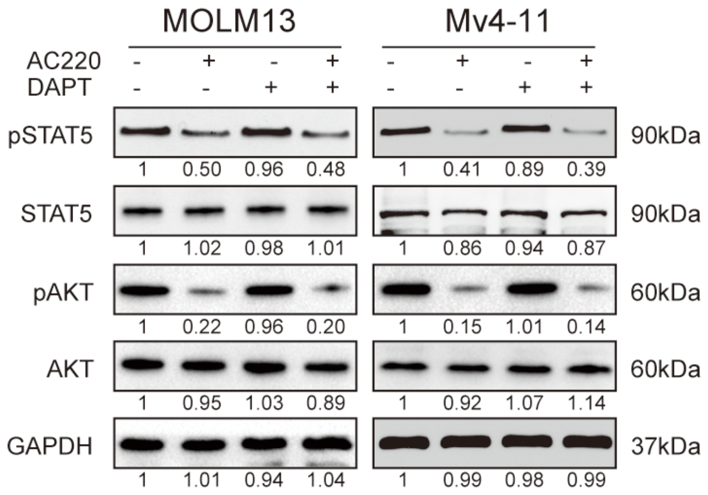

Supplemental Figure 7. Expression of downstream of FLT3

The expression of phospho-STAT5 (pSTAT5), total STAT5, phospho-AKT (pAKT) and total AKT in Molm13 and MV4-11 cells was measured by western blotting following treatment with AC220 (2.5 nM) and/or DAPT (2.5 μM) for 12 h. GAPDH was used as a loading control. Images are representative of three independent experiments.

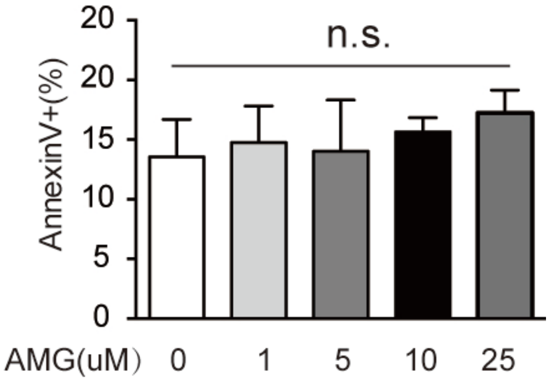

Supplemental Figure 8. AMG487 had little effect on peripheral blood mononuclear cells from healthy donors

Peripheral blood mononuclear cells obtained from healthy donors(n=4) were treated with 0~25 μM AMG487 for 48 hours and apoptosis was measured by Annexin V binding. Error bars indicate the average ± SD (ns. not significant).

Supplemental Figure 9. Representative raw plots for apoptosis assay.

Figure 1I

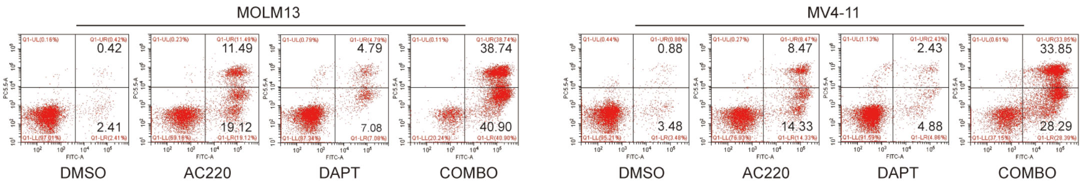

Figure 2C

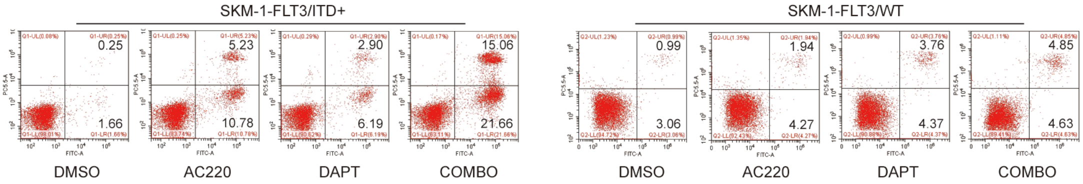

Figure 2F

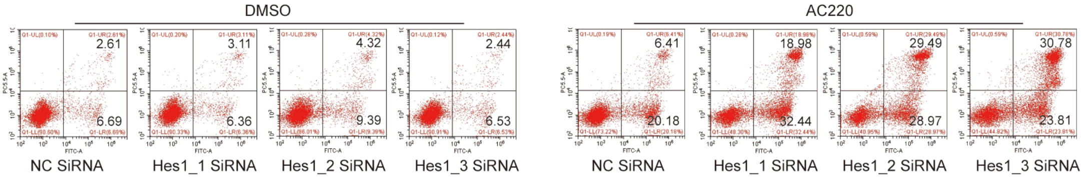

Figure 2H

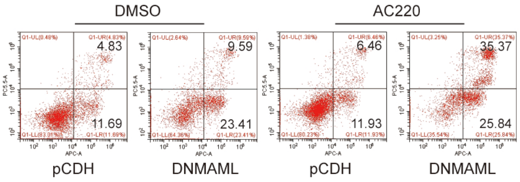

Figure 2J

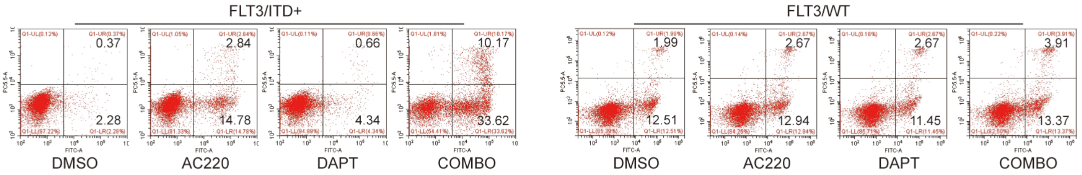

Figure 5C

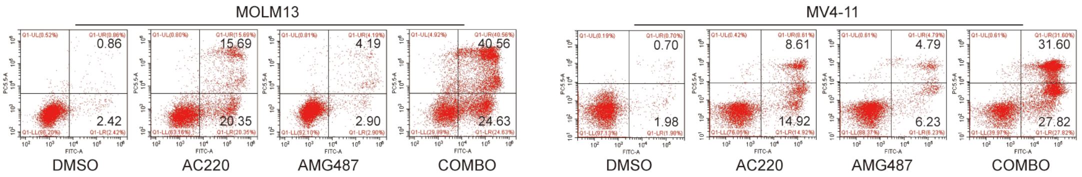

Figure 5F

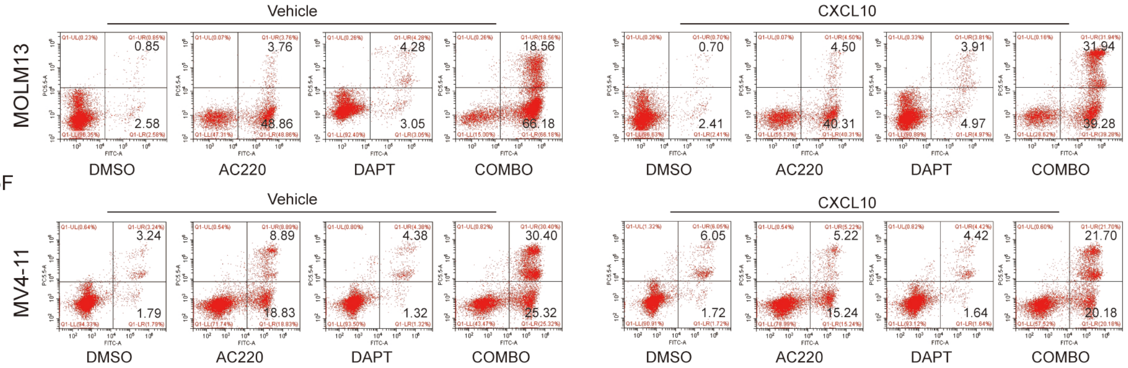

Figure 5I

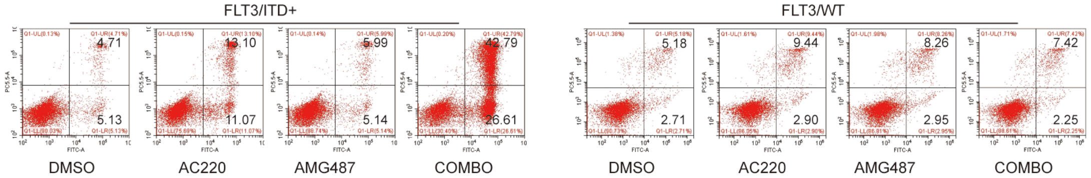

Supplementary Tables

Supplemental Table 1. AML patient characteristics

Patient ID	Age	Gender	FLT3-Mutation	Other Gene Mutation	Karyotype at baseline	Diagnostic /relapse
4	70	F	ITD		46，XX[4]	Relapse
8	45	F	ITD		46，XX[20]	Diagnostic
9	63	M	WT	PTPN11; TET2; BRAF	46，XY[20]	Diagnostic
19	35	M	WT	U2AF10;RUNX1;ETV6;BCOR;KMT2C ; FOXO1	46，XY[16]	Diagnostic
20	52	M	ITD	WT1; CEBPA	41，XY，del(1)(q24q42),-3,-4,-6,-7,-13*2,+mar[1]/46,XY,del(9)(q31)[2]/46,XY[17]	Diagnostic
21	60	F	ITD	NPM1	46，XX[20]	Diagnostic
24	48	F	ITD	NPM1; DNMT3A-R882H	46，XX[7]	Diagnostic
25	54	F	ITD	NPM1; DNMT3A; ASXL1	45,XX,11q+,-15[1]/46,XX[19]	Relapse
34	38	M	ITD	RUNX1	NA	Relapse
37	62	F	WT	CEBPA; DNMT3A; IDH1	47,XX,+8[3]/46,XX,[17]	Diagnostic
40	56	M	WT	IDH2; NPM1	46，XY[4]	Relapse
42	52	F	WT	IDH1; NPM1; NRAS; PTPN11	46，XX[20]	Diagnostic
43	51	M	ITD	DNMT3A; TET2; NPM1	46，XY[12]	Diagnostic
49	18	M	WT	CEBPA	46，XY[10]	Relapse
52	63	M	WT		46，XY[20]	Diagnostic
53	53	F	ITD	DNMT3A; NPM1	46，XX[20]	Relapse
58	61	M	WT		45,X,-Y,t(6;11)(q27;q23)[5]/46,XY[5]	Diagnostic
60	26	F	ITD		46，XX[15]	Diagnostic
66	51	M	WT	NRAS;ATM; DNMT3A; KMT2C	46，XY[12]	Diagnostic

Supplemental Table 2. Information on the oligonucleotides used in this paper

Purpose	Name	Source	sequence
Q-PCR	FLT3-Forward	TsingKe	GCAATTTAGGTATGAAAGCCAGCT
	FLT3-Reverse	TsingKe	CTTTCAGCATTTTGACGGCAACC
	MAML3-Forward	TsingKe	GAGAGTGCGAGCGTGAAGAG
	MAML3- Reverse	TsingKe	GAGAGTGCGAGCGTGAAGAG
	PSEN2-Forward	TsingKe	AGTGTGTGATGAGCGGACG
	PSEN2- Reverse	TsingKe	ACTGGGCAGTGTTCTCTCCAT
	Hes1-Forward	TsingKe	TCAACACGACACCGGATAAA
	Hes1- Reverse	TsingKe	TCAGCTGGCTCAGACTTTCA
	CXCR3-Forward	TsingKe	CCACCTAGCTGTAGCAGACAC
	CXCR3- Reverse	TsingKe	AGGGCTCCTGCGTAGAAGTT
	Hes5-Forward	TsingKe	TCAGCCCCAAAGAGAAAAAC
	Hes5- Reverse	TsingKe	TAGTCCTGGTGCAGGCTCTT
	Hey1-Forward	TsingKe	GTTCGGCTCTAGGTTCCATGT
	Hey1- Reverse	TsingKe	CGTCGGCGCTTCTCAATTATTC
	RBPJ-Forward	TsingKe	CGGCCTCCACCTAAACGAC
	RBPJ- Reverse	TsingKe	TCCATCCACTGCCCATAAGAT
	Deltex1-Forward	TsingKe	CAGTCCATGCACCAGTTTCG
	Deltex1-Reverse	TsingKe	TCCATATCGTAGGCCGTCCAT
	GADPH-Forward	TsingKe	CATGAGAAGTATGACAACAGCCT
	GADPH- Reverse	TsingKe	CTTTCAGCATTTTGACGGCAACC
Guide RNA	FLT3 gRNA-Forward	TsingKe	CACCTCTGTAGCTGGCTTTCATAC
	FLT3 gRNA-Reverse	TsingKe	AAACGTATGAAAGCCAGCTACAGA

Supplemental Table 3. Information on the reagents used in this paper

REAGENT	SOURCE	IDENTIFIER
**Antibodies**		
FITC anti-human CD34 Antibody	BioLegend	343604
FITC anti-human CD45 Antibody	BioLegend	368508
CXCR3	Abcam	Ab154845
Flt-3/Flk-2(S-18)	Santa Cruz	SC-480
Phospho-FLT3 (Tyr589/591) (30D4) Rabbit mAb	Cell Signaling Technology	3463S
Stat5 Antibody(C-17)	Santa Cruz	SC-835
Phospho-Stat5 (Tyr694) Antibody	Cell Signaling Technology	9351S
Akt (pan) (C67E7) Rabbit mAb	Cell Signaling Technology	4691S
Phospho-Akt (Ser473) Antibody	Cell Signaling Technology	9271S
p44/42 MAPK (Erk1/2) Antibody	Cell Signaling Technology	9102S
Phospho-p44/42 MAPK (Erk1/2) (Thr202/Tyr204) Antibody	Cell Signaling Technology	9101S
Cleaved Notch1 (Val1744) (D3B8) Rabbit mAb	Cell Signaling Technology	4147S
Notch2 (D76A6) XP® Rabbit mAb	Cell Signaling Technology	5732T
Notch3 (D11B8) Rabbit mAb	Cell Signaling Technology	5276T
Notch4 (L5C5) Mouse mAb	Cell Signaling Technology	2423S
Presenilin 2 (D30G3) Rabbit mAb	Cell Signaling Technology	9979S
HES1 (D6P2U) Rabbit mAb	Cell Signaling Technology	11988S
Gapdh Antibody	Proteintech	60004-1-Ig
HRP-conjugated Affinipure Goat Anti-Rabbit IgG(H+L)	Proteintech	SA00001-2
HRP-conjugated Affinipure Goat Anti-Mouse IgG(H+L)	Proteintech	SA00001-1
**Chemicals**		
AC220(Quizartinib)	SelleckChem	S1526
DAPT	SelleckChem	S2215
RO4929097	SelleckChem	S1575
AMG487	MedChemExpress	HY-15319
Sorafenib	MedChemExpress	HY-10201A
Recombinant Human CXCL10/IP-10 Protein	R&D Systems	266-IP-010
